# Reply to Zandi, M.; Soltani, S. Comment on “Alfassam et al. Development of a Colorimetric Tool for SARS-CoV-2 and Other Respiratory Viruses Detection Using Sialic Acid Fabricated Gold Nanoparticles. *Pharmaceutics* 2021, *13*, 502”

**DOI:** 10.3390/pharmaceutics14091878

**Published:** 2022-09-06

**Authors:** Haya A. Alfassam, Majed S. Nassar, Manal M. Almusaynid, Bashayer A. Khalifah, Abdullah S. Alshahrani, Fahad A. Almughem, Abdullah A. Alshehri, Majed O. Alawad, Salam Massadeh, Manal Alaamery, Ibrahim M. Aldeailej, Aref A. Alamri, Abdulwahab Z. Binjomah, Essam A. Tawfik

**Affiliations:** 1 KACST-BWH Centre of Excellence for Biomedicine, Joint Centers of Excellence Program, King Abdulaziz City for Science and Technology (KACST), Riyadh 11442, Saudi Arabia; 2 National Center for Biotechnology, Life Science and Environment Research Institute, King Abdulaziz City for Science and Technology (KACST), Riyadh 11442, Saudi Arabia; 3 National Center for Pharmaceutical Technology, Life Science and Environment Research Institute, King Abdulaziz City for Science and Technology (KACST), Riyadh 11442, Saudi Arabia; 4 Microbiology Department, Riyadh Regional Laboratory, Ministry of Health, Riyadh 12746, Saudi Arabia; 5 Material Science Research Institute, King Abdulaziz City for Science and Technology (KACST), Riyadh 11442, Saudi Arabia; 6 Developmental Medicine Department, King Abdullah International Medical Research Center, King Saud Bin Abdulaziz University for Health Sciences, King Abdulaziz Medical City, Ministry of National Guard Health Affairs, Riyadh 11481, Saudi Arabia; 7 College of Medicine, Alfaisal University, Riyadh 11533, Saudi Arabia

When COVID-19 was first announced back in 2019, there were vast number of attempts to halt the progression of the SARS-CoV-2 virus once and for all. Efforts to develop fast detection assays for this virus were under development even before we understood the structure of this virus. Several papers have revealed that SARS-CoV-2 shares the same genome sequence to those of the previously occurring coronaviruses by up to 90–99% [1,2,3,4]. Several studies have confirmed the structure of SARS-CoV-2, in which it has a relatively large positive single-stranded RNA genome, comprising highly conserved basic genes within its sequence, as follows: 5′ ORF1-HE-S-E-M-N 3′ [5,6,7,8,9,10], Upon entry of the virus, it binds to the host’s receptor.

Sialic acid (SA) is the glycoprotein that is densely projected on the surface of different mammalian cells including lung epithelial cells. It is used to mediate the binding with viral surface proteins like hemagglutinin in influenza B virus [11,12]; hemagglutinin esterase in OC43, HKU-1, and influenza C viruses [13,14]; and spike glycoprotein domain of spike protein in MERS-CoV [15,16]. Evidently, sia–virus conjugation is used to mediate the entry of different invading viruses. For example, sia–hemagglutinin in influenza B virus [11,12]. The structure of influenza B virus has two surface glycoproteins, the hemagglutinin and the neuraminidase. The hemagglutinin and NA proteins have been found to bind to SA in the host cell molecule. The hemagglutinin is known as the main envelop glycoprotein of influenza B virus, inducing receptor binding, while neuraminidase works as a separate glycoprotein. Recently, Patel et al. have investigated the effect of hemagglutinin esterase (HE) as a potential inhibitor of the emerging coronavirus utilizing a molecular docking and dynamic simulation approach. The results showed a preliminary level of HE targeting to develop effective inhibitors [5]. Some of the coronaviruses have sia–hemagglutinin esterase conjugation that mediates their entry, such as OC43 and HKU-1 viruses [13,14].

However, the role of SA in SARS-CoV-2 needs more elaboration, as the efforts of researchers to understand the full aspects of its role are still at their peak [17]. It is worth mentioning that, when SA binds to its glycoprotein counterpart, it causes red blood cells to agglutinate (hemagglutination) [18]. Hemagglutination is a reaction between the glycoprotein (i.e., hemagglutinin) presented on the surface of some enveloped viruses, such as the influenza virus, and red blood cells, causing their clumping and the formation of lattice. It could be used to quantify viruses and to detect antibodies developed against a virus through hemagglutinin inhibition (HI) assay. Hemagglutinin protein can also be used to facilitate and specify the binding with viruses, and thus can be vital in developing antiviral therapies in the future, especially using nanoparticulate systems [19,20]. However, the complexity of the hemagglutinin structure may hinder the structural characterization of this protein after binding with a specific ligand.

Nevertheless, the use of SA in this study was an attempt to bind specifically to SARS-CoV-2 virus in order to develop a fast detection tool. Having the ability to bind to other respiratory virus through the above-mentioned proteins was an additional advantage to our system that allowed us to investigate further and attempt to develop the binding of these NPs specific for each virus.

Leonardo Bò and his team illustrated the ability of SARS-CoV-2 spike protein to bind to sialic acid molecules [21]. Another study claimed that N-acetyl neuraminic acid has affinity binding to the SARS-CoV-2 spike protein via its receptor-binding domain (RBD) and the S1 N-terminal domain, demonstrating the glycan binding [22]. Therefore, sialic acid binding by the S1 spike protein engages the host cell, while S2 enhances the viral fusion [22]. Many reported that this binding is enhanced by the spread of the infection of coronaviruses because of the structure of 9-O-acetylated sialic acid binding to strain OC43 by cryo-EM [14,23]. A recent study reported that SARS-CoV-2 shares similar sequences of the spike protein with MERS-CoV, which can enter the host cells by binding to DPP4 or sialic acids. This study shows that sialic acid can interact with the spike protein of SARS-CoV-2 by binding between the two RBDs of the spike protein [21]. Furthermore, the presence of spike glycoprotein at the surface of MERS-CoV enhances the entry into the host cells. It is found that MERS-CoV mainly infects human lung epithelial cells by interacting with DPP4 and binding with α2,3-linked sialosides [15,16].

We would like to thank the referees for their thoughtful and helpful comments [24] that have undoubtedly improved the paper, for which we are very grateful. We are also ready to correct the information in the published paper. The comment has corrected highly debatable information, in which we claimed that the SA binds only to the hemagglutinin proteins rather than the S protein, especially for SARS-CoV-2 and MERS-CoV. In order to fully understand this binding, a further investigation is required. However, dealing with highly infectious viruses limits the amount of experiments that we can perform.
